# Whole genome nucleosome sequencing identifies novel types of forensic markers in degraded DNA samples

**DOI:** 10.1038/srep26101

**Published:** 2016-05-18

**Authors:** Chun-nan Dong, Ya-dong Yang, Shu-jin Li, Ya-ran Yang, Xiao-jing Zhang, Xiang-dong Fang, Jiang-wei Yan, Bin Cong

**Affiliations:** 1 Department of Forensic Medicine, Hebei Medical University, Hebei Key Laboratory of Forensic Medicine, No. 361 Zhongshan East Road, Shijiazhuang 050017, PR China; 2 Key Laboratory of Genome Sciences, Beijing Institute of Genomics, Chinese Academy of Sciences, No. 1–104 Beichen West Road, ChaoYang, Beijing 100101, PR China; 3 University of Chinese Academy of Sciences, Beijing 100049, China

## Abstract

In the case of mass disasters, missing persons and forensic caseworks, highly degraded biological samples are often encountered. It can be a challenge to analyze and interpret the DNA profiles from these samples. Here we provide a new strategy to solve the problem by taking advantage of the intrinsic structural properties of DNA. We have assessed the *in vivo* positions of more than 35 million putative nucleosome cores in human leukocytes using high-throughput whole genome sequencing, and identified 2,462 single nucleotide variations (SNVs), 128 insertion-deletion polymorphisms (indels). After comparing the sequence reads with 44 STR loci commonly used in forensics, five STRs (TH01, TPOX, D18S51, DYS391, and D10S1248)were matched. We compared these “nucleosome protected STRs” (NPSTRs) with five other non-NPSTRs using mini-STR primer design, real-time PCR, and capillary gel electrophoresis on artificially degraded DNA. Moreover, genotyping performance of the five NPSTRs and five non-NPSTRs was also tested with real casework samples. All results show that loci located in nucleosomes are more likely to be successfully genotyped in degraded samples. In conclusion, after further strict validation, these markers could be incorporated into future forensic and paleontology identification kits, resulting in higher discriminatory power for certain degraded sample types.

Chromatin organization into nucleosomes is the basal level of DNA packaging in eukaryotes. Most nucleosome research presently focuses on the relationship between some essential cellular process (such as replication, transcriptional regulation, or DNA repair) and nucleosome positioning or structure. This is because nucleosomes limit accessibility to regulatory factors^1^,^2^, and many cellular signaling events affect nucleosome composition and localization^3^,^4^,^5^. However, our research is more concerned with genetic markers, particularly short tandem repeat (STR) loci protected by nucleosomes, which may prove beneficial in the analysis of degraded DNA in forensic science, archaeology, and paleontology.

The nucleosome subunit of chromatin contains 200 bp of DNA, wound around an octamer of small, basic histone proteins into a beadlike structure. Nucleosomal DNA is divided into two regions: core DNA and linker DNA. The association of DNA with the histone octamer forms a core particle containing 147 bp of DNA. The major part of the core DNA is tightly bound about the nucleosome, whereas the terminal regions of the core and the linker regions are unbound. The core particle is very stable. An *in silico* whole human genome annotation of nucleosome exclusion regions shows that regions free of nucleosomes correlate well with DNase I hypersensitive sites, from which the inference can be made that DNA bound in nucleosomes is protected against DNases in general^6^.

Ancient DNA is extensively modified by hydrolysis and oxidation, leading to deletions, oxidized pyrimidines, and intermolecular and intramolecular cross-linking. Dixon *et al.*^7^,^8^ suggested that nucleosomes may offer protection to the 147 bp of DNA that are bound to it from endonuclease attacks, which would freely digest post-mortem DNA at exposed sites. Another ancient DNA degradation study showed that the majority of extracted ancient DNA fragments were 100–200 bp regardless of the specimen’s preservation conditions or age. The researchers postulated that the size of DNA fragments extracted from ancient sources is approximately that protected by the nucleosome core^9^.

Based on the circumstantial evidence above, we hypothesized that biomarkers in core DNA may be more protected by nucleosome structure, than are those in linker DNA. Using high-throughput sequencing techniques we assessed the *in vivo* positions of more than 35 million putative nucleosome cores in human leukocytes and indentified three types of biomarkers. We then compared nucleosome protected biomarkers with other commonly used forensic loci using artificially degraded DNA and real casework samples. Biomarkers protected by nucleosomes were better able to withstand degradation, which should allow them to be incorporated into future forensic and paleontology identification kits, particularly those dealing with ancient or otherwise degraded sample types.

## Results

### Mapping nucleosome positions by high-throughput sequencing

**Figure 1:**
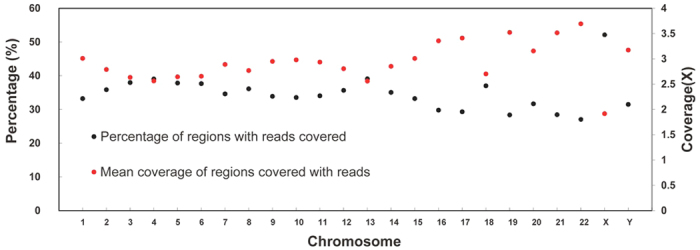
The percentage of regions with reads covered (black dots) and the mean coverage of chromosomes covered with reads (red dots).

To analyze nucleosome positioning across the genome in human leukocytes, we isolated mono-nucleosome-sized DNA from MNase-digested chromatin and sequenced the DNA ends using Solexa sequencing technology. We obtained 17,752,559 2 × 100 pair-ended reads, and ~75% of which align to unique genomic loci (Table 1). The average GC content of the reads from the nucleosome core fragment experiment is 52%, higher than the value of entire human genome, which is 42.5%. DNA fragments of high GC content are more flexible and more prone to bind with octamer into nucleosome^10^. All regions with read coverage account for about 34% of the entire genome (1,042,463,676/3,095,693,983), ranging from 27% (chr 22) to 52% (chr X), and the average sequencing depth reaches approximately 3× in these regions (Fig. 1).

**Figure 2:**
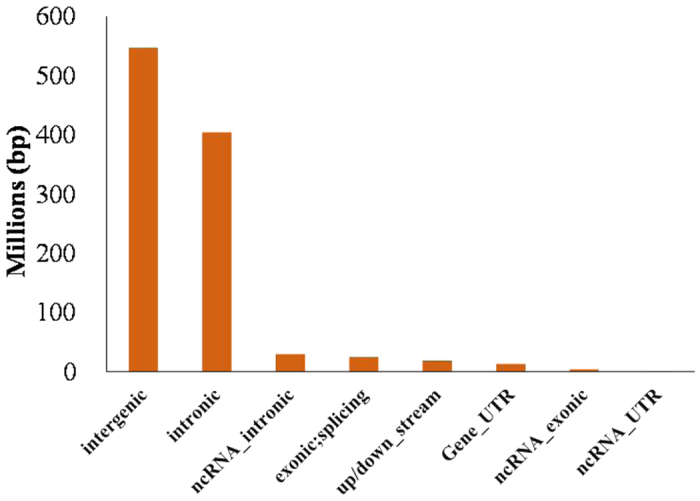
The distribution of sequenced reads across genomic regions.

The short reads obtained from sequencing were mapped to the human genome (hg19) and the distribution in the genome was analyzed. As shown in Fig. 2, the majority of well-positioned nucleosomes were detected in intergenic and intronic regions (52.5% and 38.8%, respectively), from which the forensic biomarkers are generally chosen. The distribution trend was consistent with the previously reported in the literature^11^,^12^.

### Screening of genetic markers in nucleosome regions

**Figure 3:**
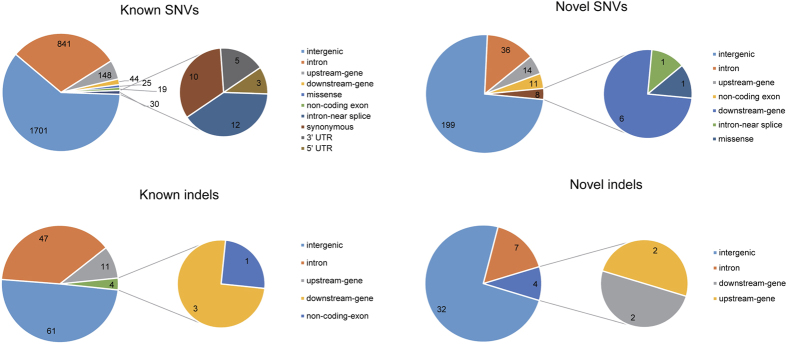
The distribution of known, novel SNVs (A,B), and known, novel indels (C,D) across genomic regions. The number of detected SNVs and indels were labled on the pie chart.

In the nucleosome-binding regions, we identified 2462 single nucleotide variations (SNVs) (Fig. 3A) and 128 indels (Fig. 3C) with at least 10 supporting reads, most of which occurred in intergenic regions. Among the detected SNVs, 2220 of them are known dbSNP variations (build 138). For the indels, 89 of them had records in dbSNP database (build 138). Some of the SNVs/indels were overlapped between different gene regions and were annotated as different DNA elements, so the total number of SNVs/indels in Fig. 3A,B was slightly higher than that of identified SNVs/indels, irrespective of known or novel ones. We had summarized all the 2462 SNVs and 128 indels into supplementary files (Text S1.xlsx and Text S2.xlsx). We compared our direct sequence reads from nulceosome core regions with 44 commonly used forensic STR loci (Text S3.xlsx). Five of these STRs (TH01, TPOX, D18S51, DYS391, and D10S1248) in our reads matched the records of 44 commonly used forensic STR loci.

### Analyzing loci relative quantity by real-time PCR

Primers should not contribute to relative quantity differences after successfully being optimized (96.818–110.185% efficiencies and 0.970–0.992 r^2^-values). However, size differences between the amplicons can affect the experiment, as shorter amplicons tend to survive degradation better^13^. Therefore, we used the Mann-Whitney *U* test to analyze amplicon length differences between the NPSTR group and non-NPSTR group. Results show no differences between these two groups (*P* values of five samples are 0.188, 0.258, 0.449, 0.060 and 0.059 respectively. All the *P* values are more than 0.05).

**Figure 4:**
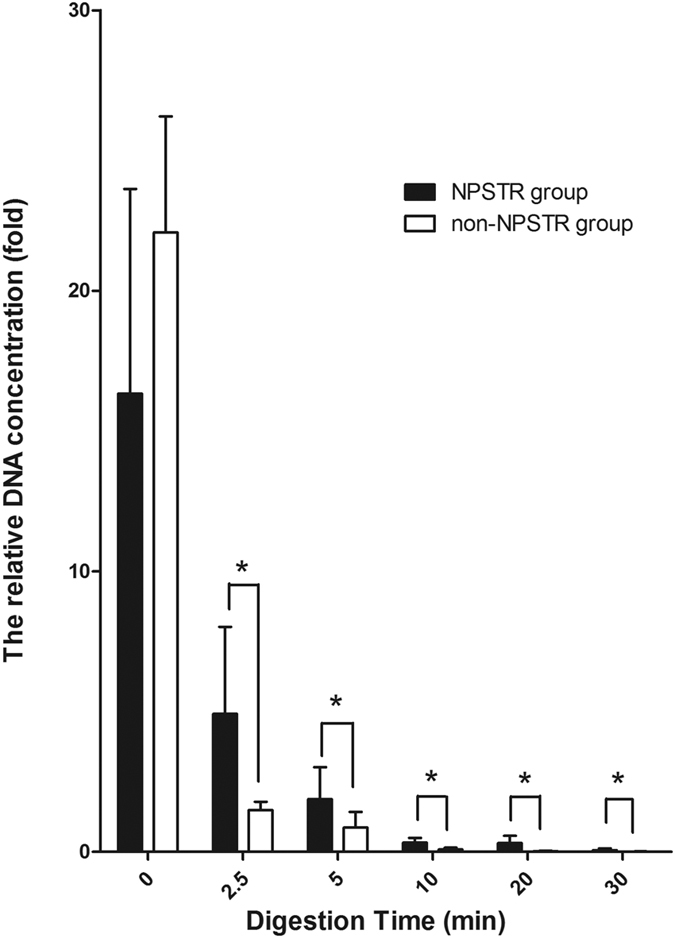
The relative DNA quantification of representative sample at each time point. The figure was generated by the normalized, individual data point 2^−ΔCT ^43. The normalized, individual data point 2^−ΔCT^ at the time point 0 min was used as benchmark, the results of other time points were compared with the benchmark. The multivariate analysis of variance process of the general linear model showed there was a significant difference between the two groups at the time points labeled with asterisks (*P* < 0.05). The representative sample A at the time points 2.5, 5, 10, 20, and 30 min, has corresponding *P* values of 0.005, 0.028, 0.001, 0.004, and 0.025 respectively.

**Figure 5:**
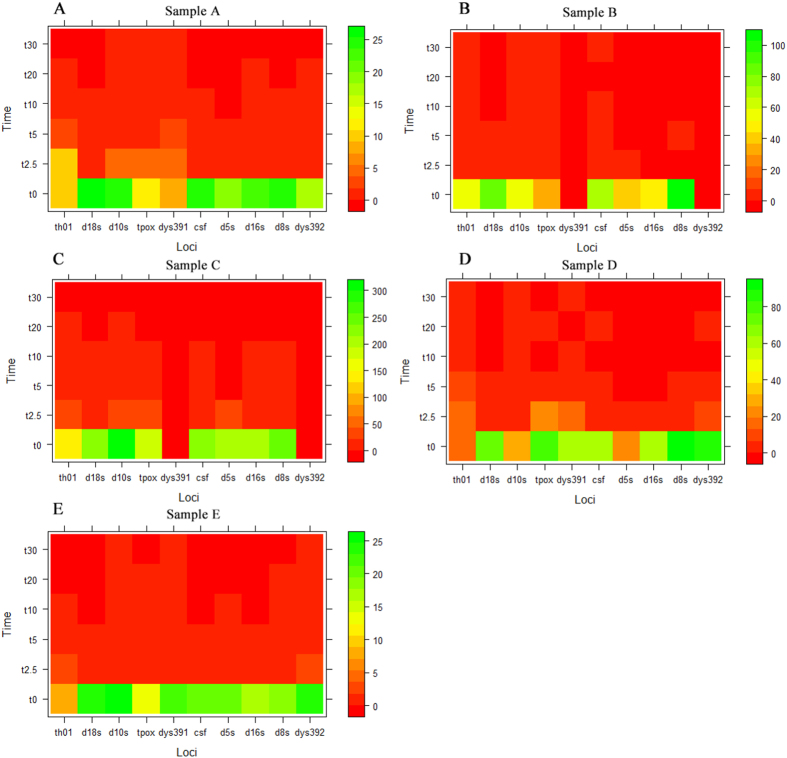
Heatmap representations of DNA concentration of 10 loci (5 NPSTRs and 5 non-NPSTRs) in 5 samples. (**A**–**E**) represent the five samples which were assessed by real-time PCR. Each row represents a time period of nuclease incubation. Each column represents a locus, the order of markers is: TH01, D18S51, D10S1248 TPOX,DYS391(5 NPSTRs) and CSF1PO, D5S818, D16S539, D8S1179, DYS392(5 non-NPSTRs). The colours are skewed into cold green-yellow for high concentration and hot orange-red for low concentration. Better performance is observed in NPSTR group compared with non-NPSTR group in all 5 samples.

Degraded samples exhibited a reduced DNA concentration with increasing incubation time in all five samples (Fig. 4). We used repeated measures and multivariate analysis of variance (MANOVA) process from the general linear model in SPSS and giving comparison among different groups and different measure time pairwise. The results show there are significant differences between the NPSTR group and the non-NPSTR group in all samples at three time points (5, 10, and 20 min) (Table 2, Fig. 4). It suggests that during our sample degradation to a certain degree (10 μg of the extracted DNA with 0.01 U/μl DNase I for time periods of 5, 10, and 20 min), the protective capability of nucleosomes probably exists. We consider the different digestion efficiency of different samples by DNase enzyme to be the main reason no difference was seen for some samples (samples B, D, and E) at two time points (2.5 and 30 min) between the two groups. The heatmap DNA concentration of 10 loci(5 NPSTRs and 5 non-NPSTRs) in 5 samples are visualized in Fig. 5. Better performance is observed in NPSTR group compared with non-NPSTR group in all 5 samples.

### Typing of artificially degraded DNA

**Figure 6:**
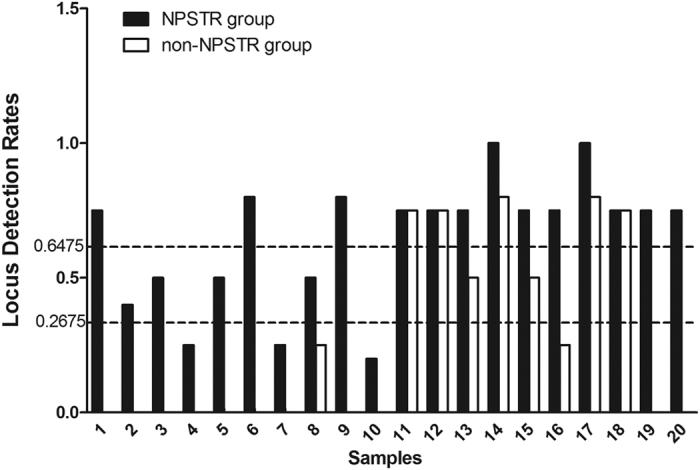
The locus detection rates in the nucleosome group and the non-nucleosome group from artificially degraded samples. The average locus detection rates of these two groups are marked with dotted lines.

Genotyping analysis of 20 artificially degraded blood samples by capillary gel electrophoresis was performed, to further validate the protective capabilities of nucleosomes. All amplifications were triplicated. When a locus to be seen in 2 of the 3 replicates, we defined that the locus is detected. And the results are summarized in Table 3. We compared the genotyping results between the non-degraded and 20 minute degraded DNA (0 min and 20 min) from the blood samples to make clear whether the alleles detected actually match the expected genotype from the individual or not. The locus detection rates after comparing is given in Fig. 6. The average locus detection rates of the NPSTR group and the non-NPSTR group are marked with dotted lines in Fig. 6. The results further confirm the protective capabilities of nucleosomes on STRs. In 20 blood samples the average locus detection rates of the NPSTR group is significantly higher than the non-NPSTR group (NPSTR group: 64.75%, non-NPSTR group: 26.75%, *P* = 0.001 < 0.05).

**Figure 7:**
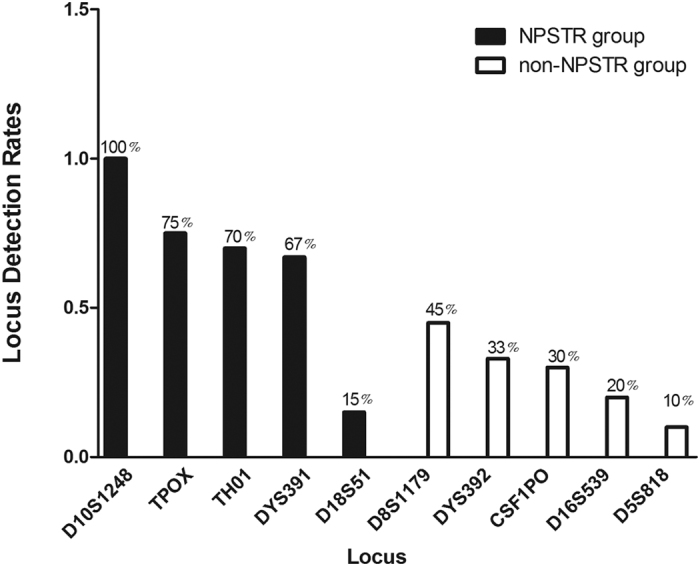
The single locus detection rates detected in 20 artificially degraded DNA samples.

The detection rates of every single locus are summarized in Fig. 7. Among the ten miniSTRs, D10S1248 of the NPSTR group was most likely to be detected by capillary gel electrophoresis (100% detected in 20 artificially degraded blood samples). D18S51 is the only locus from the NPSTR group whose detection rate was less than 60%. We consider that the relatively long amplicon size of D18S51 may contribute to this result. We think with the reduction of all the allelic amplicons of locus D18 in further experiment, the detection rate of D18S51 will increase than it does now. The single locus detection rates of the non-NPSTR group was low, varying from 10% to 45%. We conclude that the four NPSTR loci (TPOX, TH01, D10S1248, and DYS391) could be well suited for profiling degraded DNA because of the intrinsic structural properties of DNA in chromatin, and three of these four loci also had good performance in degraded DNA analysis in previous report, such as TPOX and TH01 still had high intensity than other loci(CSF1PO, FGA, D21S11,and D7S820) for the sample exposed to the highest temperature after 84 days^14^. Another report showed that DYS391 had good performance for the 120-year-old skeletal remains of Ezekiel Harper comparing with the other loci of AmpFlSTR Yfiler^®^ after three different DNA extraction methods^15^.

### Typing of different tissue samples

We performed genotyping analysis on 3 type of tissue samples with 10 miniSTRs (NPSTRs and non-NPSTRs) including epithelial tissue, hair root tissue and cartilage tissue. All the samples were amplified in triplicate and the results are summarized in Text S4.xlsx.

**Figure 8:**
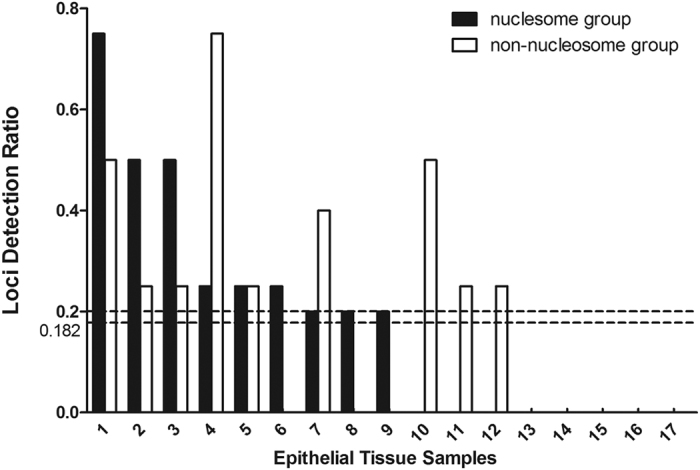
The locus detection rates in the nucleosome group and the non-nucleosome group from artificially degraded epithelial tissue samples. The average locus detection rates of these two groups are marked with dotted lines.

**Figure 9:**
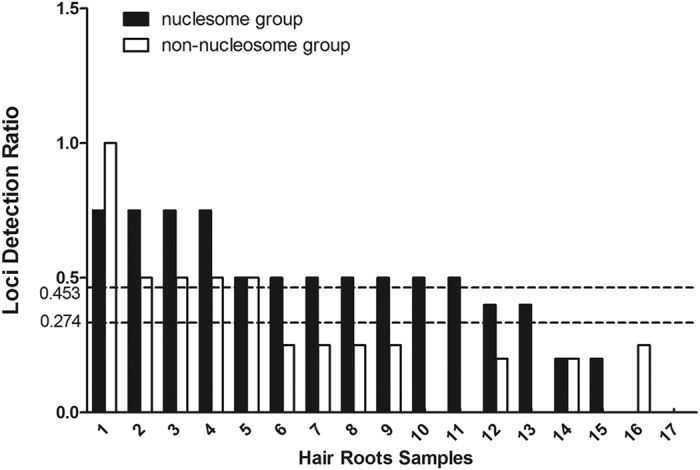
The locus detection rates in the nucleosome group and the non-nucleosome group from artificially degraded hair root tissue samples. The average locus detection rates of these two groups are marked with dotted lines.

**Figure 10:**
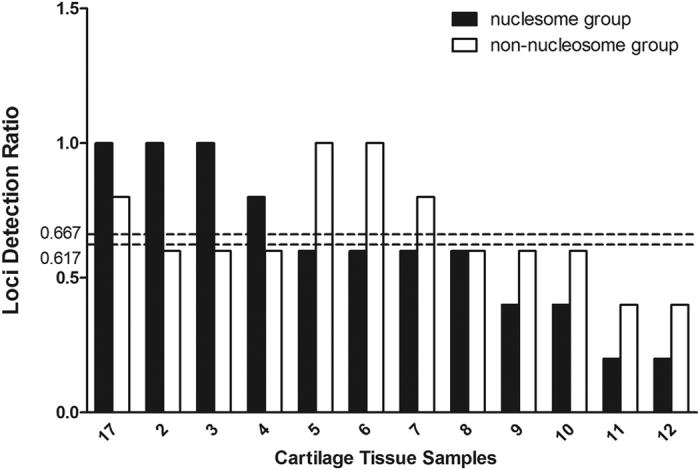
The locus detection rates in the nucleosome group and the non-nucleosome group from artificially degraded cartilage tissue samples. The average locus detection rates of these two groups are marked with dotted lines.

The locus detection rates after comparing the genotyping results of different degraded DNAs (0 min and 20 min) from the epithelial tissue, hair root tissue and cartilage tissue samples are given separately in Figs 8, 9, 10. The average locus detection rates of the NPSTR group and the non-NPSTR group are marked with dotted lines in each Figure. In hair root tissue samples the average locus detection rates of the NPSTR group is significantly higher than the non-NPSTR group (NPSTR group: 45.30%, non-NPSTR group: 27.40%, *P* = 0.011 < 0.05). While in epithelial tissue and cartilage tissue samples, there is no differences between the NPSTR group and the non-NPSTR group (*P*_epithelial tissue_ = 0.70 > 0.05, *P*_cartilage tissue_ = 0.629 > 0.05).

### Typing of casework samples

We performed genotyping analysis on the 12 formalin fixed samples with 10 miniSTRs (NPSTRs and non-NPSTRs). All the samples were amplified in triplicate and the results are summarized in Text S5.xlsx. Because every four tissues were selected from the same individual, exact alleles could be detected by comparison to reference and consensus genotypes.

**Figure 11:**
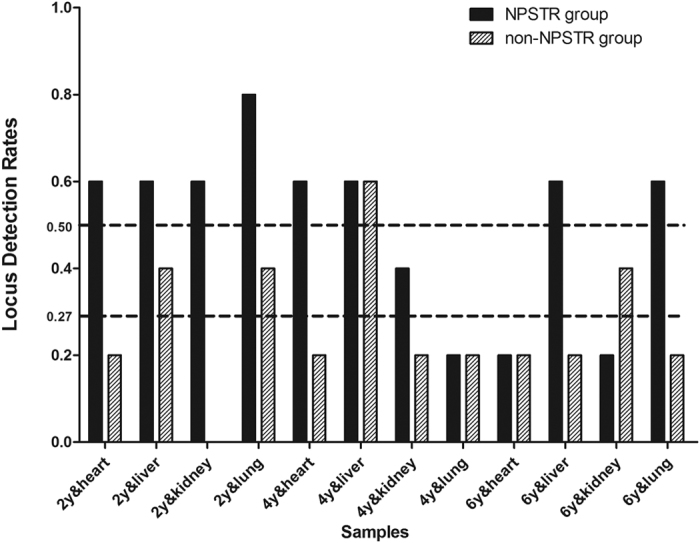
The locus detection rates in the nucleosome group and the non-nucleosome group from formalin fixed samples. The average locus detection rates of these two groups are marked with dotted lines.

**Figure 12:**
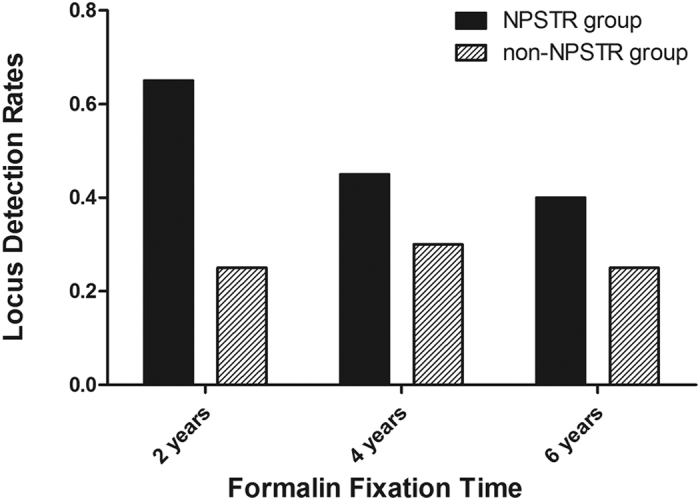
The locus detection rates for different fixation times in the nucleosome group and the non-nucleosome group from formalin fixed samples.

The locus detection rate for each formalin fixed sample detected by the 10 miniSTRs is shown in Fig. 11. The average locus detection rates of these two groups are marked with dotted lines. For all 12 samples the average locus detection rates of the NPSTR group was 50%; however, the values for the non-NPSTR group were 27%. The value of the NPSTR group was significantly higher than that of the non-NPSTR group (*P* = 0.008 < 0.05). We further analyzed the effect of different fixation times on the detection rates (Fig. 12). There was a downward trend of detection rates with increasing fixation times in NPSTR group, but regardless of fixation time length, the loci detection rates for the NPSTR group is always higher than that for the non-NPSTR group.

## Discussion

DNA identification techniques are primarily based on the determination of the size or sequence of desired PCR products. The fragmentation of DNA templates or structural modifications that occur during decomposition can impact the outcome of analytical procedures. Conventional methods for analyzing degraded DNA include increasing the sensitivity of the PCR reaction, using short amplicons designs, such as mini-STR^16^ and as well as using multi-copy markers, such as mtDNA. These strategies had proven successful for obtaining more informative DNA profiles with degraded DNA samples, such as those often found in mass disasters and samples exposed to extreme environments. However, a new strategy based on taking advantage of the intrinsic structural properties of DNA in chromatin, such as nucleosomes structures, could offer protection to bound DNA by limiting access to enzymes. This strategy could be applied in practice to obtain better DNA profiles from degraded samples.

The long DNA strands of every eukaryotic cell’s genome are packaged into chromatin in a very confined nuclear volume^17^. The initial idea for our research was based on the folded, three-dimensional structure of DNA in the cell, especially the nucleosome structure, which may provide the potential benefit of resistance to DNA fragmentation. At the same time, several circumstantial evidences reported in bioinformatics^6^, biochemistry^7^,^8^, and archaeology^9^ also support our idea.

In the present study we aim to experimentally verify the nucleosome protection hypothesis by discovering STRs within nucleosome core regions, using whole genome sequencing, and to demonstrate the potential benefit of resistance to several common degradation processes provided by the persistence of histone-DNA complexes. Although a few previous studies have tested this hypothesis, unfortunately these studies^18^,^19^,^20^ were all based on computerized prediction and resulted in controversial results. Thanakiatkrai’s research^19^ concluded that nucleosome protection did not exist for degraded saliva samples, given that the software nuScores accurately represents the probability of finding a nucleosome. While Freire-Aradas’s research^20^ showed the nucleosome single nucleotide polymorphism (SNP) assays gave genotyping success rates 6% higher than the best existing forensic SNP assay; *e.g.* the SNP identification for ID Auto-2 29- plex and significantly higher than the miniSTR assay based on the software RECON.

Many factors can affect the formation and location of nucleosomes, and the associations of DNA with histones, including dinucleotide periodicity and stacking, GC content, and chromatin remodelers^6^,^21^,^22^,^23^,^24^,^25^,^26^. Thanakiatkrai *et al.*^19^ explored two nucleosome positioning signals (DNA bendability based on known stiff sequences and dinucleotide base stacking) via two computer programs to select nucleosome loci. These signals only indicate the probability of finding a nucleosome at a given location; other nucleosome location factors might be ignored. Thus, the whole genome sequencing-based mapping of nucelosome positions conducted in our study should provide more accurate and direct results compared with computerized prediction methods.

Although a variety of nucleosome positioning maps have been described for single cell types of human promoters and other human genomic regions^11^,^27^,^28^, a genome-wide map of nucleosomes in the human leukocyte has not yet been reported. Blood is the main material for forensic identification. The nuclear DNA is from the karyote (mainly the human leukocyte) of the blood sample. Human leukocytes as the main source of DNA for forensic identification include five different and diverse cell types. More complex multicellular systems with multiple cell types provide a number of interesting challenges and opportunities for nucleosome positioning study. In our study, human leukocytes were used to perform a nucleosome positioning study that not only provides nucleosome position information in the human leukocyte, but that can also make the selected biomarkers more in line with practical applications in forensic science, archeology, and paleontology.

Nucleosome positioning is heavily tissue dependent. In order to test the protective capabilities of 5 NPSTRs in different types of tissue, we performed genotyping analysis on 3 type of tissue samples with 10 miniSTRs (NPSTRs and non-NPSTRs) including epithelial tissue, hair root tissue and cartilage tissue. The results showed that in hair root tissue samples the average locus detection rates of the NPSTR group is significantly higher than the non-NPSTR group which was the same as in the blood samples. While in epithelial tissue and cartilage tissue samples, there is no differences between the NPSTR group and the non-NPSTR group. We think that the different nucleosome positioning in different types of samples is the main reason.

In our study, the locus detection rates of the NPSTR group between artificially degraded blood samples and formalin fixed case samples were 64.75% and 50%, respectively. However, the values of the non-NPSTR group between these two kinds of samples showed almost no differences (26.75% and 27% respectively). This may have been due to the influence of factors on the DNA in formalin fixed case samples being more complex than in our artificially degraded samples. Presently 10% formalin remains the most widely used fixative by hospitals and research organizations for fixing human tissues^29^. This process can result in highly degraded DNA due to physical and chemical influence factors, such as fixation time^30^, the thickness of the slice, extensive DNA crosslinking, and formaldehyde in the fixative^29^. It is usually very difficult to get more informative genotypes with such samples. Our results show that the formalin fixed case samples have average locus detection rates of the NPSTR group significantly higher than that of the non-NPSTR group (*P* = 0.008 < 0.05).

Degraded DNA sources are not particularly limiting regarding the quantity of material available for amplification. Therefore, based on our experimental confirmation that nucleosomes confer protection that can provide better DNA profiles from degraded samples, choosing and incorporating STRs that are protected by nucleosomes could be recommended as a new strategy for analyzing degraded DNA. Meanwhile due to advantages of SNPs and INDELs (can provide population data information in dbSNP database; can easily be extrapolated from the 1000 genomes project dataset; include other information ancestral informativeness for instance), Nucleosome -SNPs and Nucleosome - INDELs will be added into further research. After further optimization, nucleosome positioning in different types of tissue, genetic investigation, and forensic parameter evaluation, the valuable loci information obtained by whole genome nucleosome sequencing could be used to develop a kit suitable for individual identification and paternity testing in forensic science and family origin tracing in the field of archaeology.

## Methods

### Isolation of mono-nucleosome core DNA fragments

The leukocytes were isolated from the peripheral blood of healthy donors (Department of HeBei Blood Center) using blood cytolysate (Solarbio, China). We used an optimized micrococcal nuclease (MNase) digestion protocol according to references^31^,^32^. The leukocytes were resuspended in 0.5% Triton/Buffer A (340 mM sucrose, 15 mM Tris at PH 7.4, 15 mM NaCl, 60 mM KCl, 2 mM EDTA, 0.5 mM EGTA, 0.2 mM PMSF, and 15 mM β-mercaptoethanol) and mixed by pipetting. After thawing on ice for 10 min, the cells were quickly and gently washed three times with Buffer A. CaCl_2_ and micrococcal nuclease (Fermentas, Lithuania, 300 U/μl) were added for final concentrations of 1 mM and 3 U/μl, respectively, followed by incubation at 37 °C for 3 h to liberate the mononucleosome cores. The reaction was stopped by adding 500 μl of 100 mM EDTA. Proteins were removed by treatment with 4 μl proteinase K (10 mg/ml in TE at pH 7.4) and 40 μl of 10% SDS for 3 h at 56 °C, followed by phenol, phenol/chloroform, and chloroform extractions, and ethanol precipitation. After RNase treatment and phenol/chloroform and chloroform extraction, separation of the MNase-digested DNA into mono-, di-, tri-, and multi-nucleosome DNA was achieved with 2% agarose gel electrophoresis, and the band corresponding to mono-nucleosomes was gel-extracted using a SanPrep Column DNA Gel Extraction Kit (Sangon Biotech, China) following the standard protocol.

### Mono-nucleosome core DNA fragments sequencing and data analysis

The DNA library was constructed using an Illumina DNA-Seq library preparation kit, according to the manufacturer’s instructions. Sequencing was then performed using the Illumina Hiseq 2000 sequencing system in a pair-ended manner with read lengths of 100 bp. Sequenced reads were aligned to the human genome (hg19) using the Burrows-Wheeler Aligner (BWA v.0.6.2-r126)^33^ with no more than three mismatches allowed for each read. Next, Samtools^34^ and VarScan^35^ were used to call genomic variants. SeattleSeq (http://snp.gs.washington.edu/SeattleSeqAnnotation138/) and ANNOVAR^36^ were used for single nucleotide variation (SNV) and insertion/deletion (Indel) annotation. We analyzed short tandem repeats (STRs) in the reads from the nucleosome core regions by comparing the sequence reads with 44 STR loci commonly used in forensics (Text S3.xlsx). Data on these loci were obtained from the Short Tandem Repeat DNA Internet DataBase (http://www.cstl.nist.gov/strbase/) and the NCBI Human Genome Map (http://www.ncbi.nlm.nih.gov/projects/genome/guide/human/).

### Artificially degraded simulated samples and case samples

Two types of samples were prepared for validating the protection ability of NPSTRs obtained by whole genome nucleosome sequencing in our study including artificially degraded simulated DNA samples and formalin fixed samples. There are 4 types of tissue samples were prepared for artificially degraded simulated DNA samples including blood tissue samples, epithelial tissue samples, hair root tissue samples and cartilage tissue samples.

All artificially degraded simulated DNA samples were genotyped by five NPSTRs and five non-NPSTRs. Five of the blood artificially degraded simulated DNA samples were selected to be used with real-time polymerase chain reaction (PCR) analysis. All formalin fixed samples were genotyped by five NPSTRs and five non-NPSTRs. All participants signed informed consent contracts, before beginning the study. This study passed the ethical review and approved by HeBei Medical University Biomedical Ethics Committee. The methods were carried out in accordance with the approved guidelines. Specific methods follow.

### Preparation of artificially degraded simulated DNA

Whole blood samples (N = 20, including 6 male samples and 14 female samples) were extracted using a QIAamp^®^ DNA Blood Midi kit (Qiagen, Germany). DNA quantification was performed using a NanoDrop 1000 spectrophotometer (Thermo Scientific, USA) as per references^37^,^38^. Whole epithelial tissue samples (N = 17, including 4 male samples and 13 female samples) and hair root tissue samples (N = 17, including 4 male samples and 13 female samples) were extracted using using the Chelex-100 method. Whole cartilage tissue samples (N = 12 male samples) were extracted using using the QIAamp^®^ DNA Investigator Kit(Qiagen, Germany).DNA quantification was performed using a NanoDrop 1000 spectrophotometer (Thermo Scientific, USA). Degraded DNA samples were prepared by enzymatically digesting 10 μg of the extracted DNA with 0.01 U/μl DNase I (Fermentas, Lithuania) for time periods of 0, 2, 5, 10, 20, and 30 min.

### Casework samples

Formalin fixed samples are commonly used for disease diagnosis and scientific research in clinical pathology and forensic pathology, among other fields. 12 formalin fixed samples (Identification Center of Forensic Medicine, HeBei Medical University) were assessed across five NPSTRs and five non-NPSTRs. The formalin fixed samples included heart, liver, lung, and kidney tissues from three male individuals, and were formalin fixed for 2, 4, or 6 years. Whole formalin fixed samples were extracted using a TIANGEN® TIANamp FFPE DNA Kit (Tiangen Biotech, China). DNA quantification was performed using a NanoDrop1000 spectrophotometer (Thermo Scientific, USA).

### Real-time PCR

A total of 30 artificially degraded DNase I treated samples were prepared. The samples consisted of five blood samples (2 female samples and 3 male samples) incubated for six time intervals (0, 2, 5, 10, 20, and 30 min). At each time point the exonuclease was inactivated, and then the DNA were amplified of selected loci (five NPSTRs and five non- NPSTRs). Primers were designed according to references^37^,^39^,^40^,^41^ and the website (http://www.cstl.nist.gov/div831/strbase/index.htm) to decrease STR amplicon size and to make sure the availability of the primers. In view of the complex nucleosome positioning in human leukocytes (multicellular systems), we designed primers by comprehensively considering the factors such as motif, primer amplification efficiency, amplion length and so on. Detailed primer information is shown in Table 4.

20 μl reaction volumes, consisting of 10 μl 2 × SYBR® Premix Ex Taq (Takara, Japan), 2 μl sample DNA, 4 pM forward and reverse primers separately, 0.4 μl 50 × ROX Dye II, and Milli-Q water (Millipore, USA). 9948 Male DNA (Promega, USA)was used as a PCR positive control and was amplified on every plate. Thermal cycling conditions follow: initial denaturation at 95 °C for 30 s, 95 °C for 5 s, and 60 °C for 34 s. A dissociation curve analysis was also carried out. Data were collected on an Applied Biosystems 7500 Real-Time PCR System. SYBR signals were normalized to ROX (reference dye).

### Capillary gel electrophoresis

All of the artificially degraded samples from 20 individuals digested at 0 min and 20 min were amplified using ten miniSTR primers with the same fluorescently labeled forward primer (6-FAM). PCR was performed in 20 μl reaction volumes, containing 4 pM of primers, 1 unit Taq DNA polymerase, 1.5 mM MgCl^2^, 1 × PCR Buffer and 10 ng DNA. The temperature profile was: 5 min at 95 °C followed by 30 s at 95 °C, 75 s at 57 °C and 45 s at 72 °C for 28 cycles and a final extension of 30 min at 72 °C. The same amplifications were performed with all the formalin fixed samples.

All amplifications were triplicated. The PCR products were separated electrophoretically using an ABI 310/3130 Genetic Analyzer, and fragment size and genotype were analyzed using GeneMapper 3.2 software. Genotyping failure was declared when no peaks were observed above the interpretational threshold of 50 relative fluorescent units (RFUs). When a locus to be seen in 2 of the 3 replicates, we defined that the locus is detected. Genotyping performance was assessed by recording locus detection rate(locus detection rates = 1 − locus dropout rates). For simplicity, we defined that homozygote and allelic drop-out both were identified as locus detected.

### Statistic analysis

Results of real-time PCR were estimated by repeated measure and a multivariate analysis of variance (MANOVA) process from the general linear model in the Statistical Package for the Social Sciences (SPSS). Results were given comparison among different groups and different measure time pairwise, as per reference^42^.

Locus detection rates between the two groups (the NPSTR group versus the non-NPSTR group) and single locus detection rates were calculated along with the typing results for the 20 artificially degraded DNA samples. A Mann-Whitney *U* test was performed to compare the locus detection rates between the two groups. The same analysis was performed for formalin fixed case samples.

## Additional Information

**How to cite this article**: Dong, C.- *et al.* Whole genome nucleosome sequencing identifies novel types of forensic markers in degraded DNA samples. *Sci. Rep.* **6**, 26101; doi: 10.1038/srep26101 (2016).

## Supplementary Material

Supplementary Text S1

Supplementary Text S2

Supplementary Text S3

Supplementary Text S4

Supplementary Text S5

## Figures and Tables

**Table 1:** The summary of sequenced reads in nucleosome core regions.

	fastq1	fastq2
Total Sequences	17,752,559	17,752,559
Sequence length	101	101
%GC	52	52
#reads map to unique loci	26,750,300 (75.34%)	
#reads map to multiple loci	1,333,728 (3.76%)	

**Table 2:** Repeated measures and multivariate analysis of variance (ANOVA) of 5 degraded samples.

sample	Mauchly’s Test of Sphericity	multivariate ANOVA and Greenhouse-Geisser	comparison between NPSTR group and non-NPSTR group at each time point					
			0	2.5	5	10	20	30
A	0.000	0.000	0.057	0.005	0.028	0.001	0.004	0.025
B	0.000	0.001	0.676	0.258	0.029	0.048	0.041	0.063
C	0.000	0.002	0.121	0.037	0.009	0.016	0.043	0.445
D	0.000	0.000	0.479	0.192	0.038	0.022	0.022	0.008
E	0.000	0.000	0.244	0.382	0.047	0.000	0.017	0.323

**Table 3:** The loci typing of blood artificial degraded DNA between NPSTR group and non-NPSTR group at the time point of 20 minutes.

Gender	DYS391	D18S51	TH01	D10S1248	TPOX	DYS392	D8S1179	CSF1PO	D5S818	D16S539	the locus detection rates of NPSTR group	the locus detection rates of non-NPSTR group
female	–	–	7,9	16,14	8	–	–	–	–	–	0.75	0
male	–	–	7,10	15	–	–	–	–	–	–	0.4	0
female	–	–	–	14,15	8	–	–	–	–	–	0.5	0
female	–	–	–	14	–	–	–	–	–	–	0.25	0
female	–	–	7,9	15,16	–	–	–	–	–	–	0.5	0
male	8	–	7,9	15	8,11	–	–	–	–	–	0.8	0
female	–	–	–	14,15	–	–	–	–	–	–	0.25	0
female	–	–	–	14,16	8	–	16	–	–	–	0.5	0.25
male	8	–	9,7	14,16	8	–	–	–	–	–	0.8	0
male	–	–	–	14,16	–	–	–	–	–	–	0.2	0
female	–	–	8,10	14	8,11	–	11,13	11,12	–	10,11	0.75	0.75
female	–	–	6,9	14,15	8,9	–	13,14	9,11	–	9,10	0.75	0.75
female	–	–	7	14,16	8,11	–	15,19	9,10	–	–	0.75	0.5
male	8	13	8,9	13,14	8,11	11	13,14	10,11	–	8,11	1	0.8
female	–	–	7,10	15,16	8	–	11,16	–	12	–	0.75	0.5
female	–	–	5	14,15	8,11	–	11,12	–	–	–	0.75	0.25
male	8	15,21	6,7	14,16	8,9	12	15	10,9	11	–	1	0.8
female	–	14,18	–	14,16	11	–	14,17	10,9	–	11,7	0.75	0.75
female	–	–	9	12,14	8,11	–	–	–	–	–	0.75	0
female	–	–	9,10	15	8,11	–	–	–	–	–	0.75	0
	0.67	0.15	0.7	1	0.75	0.33	0.45	0.30	0.10	0.20	single locus detection rates	

**Table 4:** Primer information of ten miniSTR.

group	Locus	Mini-STR primers (5′–3′)	Allele range	Mini-STR size (bp)
nuclosome group	TPOX	**F**- AGGCACTTAGGGAACCCT	6–13	64–92
		**R**-GTCAGCGTTTATTTGCCC		
nuclosome group	D18S51	**F**-TGAGTGACAAATTGAGACCTT	7–27	113–193
		**R**-GTCTTACAATAACAGTTGCTACTATT		
nuclosome group	TH01	**F**-CCTGTTCCTCCCTTATTTCCC	3–14	51–98
		**R**-GTTTCTT GGGAACACAGACTCCATGGTG		
nuclosome group	D10S1248	**F**-TTAATGAATTGAACAAATGAGTGAG	8–19	79–123
		**R**- GCAACTCTGGTTGTATTGTCTTCAT		
nuclosome group	DYS391	**F**- TTCAATCATACACCCATATCTGTC	6–14	89–121
		**R**-GATAGAGGGATAGGTAGGCAGGC		
non-nucleosome group	D8S1179	**F**-GTATTTCATGTGTACATTCG	8–19	68–112
		**R**-GATTATTTTCACTGTGGGG		
non-nucleosome group	D16S539	**F**-CAGACAGACAGACAGGTG	8–15	86–114
		**R**-GTATCTATCATCCATCTCTG		
non-nucleosome group	CSF1P0	**F**-CAGTAACTGCCTTCATAGATAG	6–15	82–118
		**R**-GACCCTGTTCTAAGTACTTCC		
non-nucleosome group	D5S818	**F**-CCTCTTTGGTATCCTTATGT	7–16	84–120
		**R**-CTTTATCTGTATCCTTATTTATACC		
non-nucleosome group	DYS392	**F**-AAAAGCCAAGAAGGAAAACAAA-3′	6–17	93–125
		**R**-GAAACCTACCAATCCCATTCCTT-3′		
